# Impact of natural biostimulants on performance, carcass characteristics, serum biochemistry, and economic effectiveness in broilers

**DOI:** 10.5194/aab-69-77-2026

**Published:** 2026-01-28

**Authors:** Sabry A. A. El-Sayed, Ahmed A. Allam, Seham M. Ibrahim, Mohamed S. A. Attia, El-Sayed I. Hassanein, Hassan A. Rudayni, Abdullah S. Alawam, Mahmoud Alagawany

**Affiliations:** 1 Department of Nutrition and Clinical Nutrition, Faculty of Veterinary Medicine, Zagazig University, Zagazig, Egypt; 2 Department of Biology, College of Science, Imam Mohammad Ibn Saud Islamic University (IMSIU), Riyadh 11623, Saudi Arabia; 3 Department of Poultry, Faculty of Agriculture, Zagazig University, Zagazig 44511, Egypt

## Abstract

In poultry production, biostimulants can replace antibiotics. These substances, made from plant extracts, improve animal growth and performance. This experiment was carried out to investigate the impact of biostimulants (NUQO©NEX) as a natural growth promoter on growth parameters and economic efficiency measurements. Unsexed 1 d old chicks ( n=90 ) of a commercial meat type (Ross 308) were randomly equally allocated into three groups of six replicates (five chicks per replicate) in this study. Group G1 fed on the control diet (without additive), while groups G2 and G3 fed on the diet supplemented with 100 mg NUQO©NEX kg^−1^ (on top and (matrix value; this matrix reduced the nutrient requirement by 0.3 % crude protein and 30 kcal metabolizable energy, respectively) for 35 d. Results showed that broiler chicks of groups G2 and G3 showed a significant ( P<0.05 ) improvement in the average feed conversion ratio (FCR) of broiler chicks compared to the control group. Group G2 recorded the best value of FCR compared to group G3 and the control group. Cholesterol and low-density lipoprotein (LDL) levels in broiler serum were decreased ( P<0.05 ) in group G3 and then in group G2 when compared to the control group. Groups G2 and G3 recorded significant ( P<0.05 ) improvements in the antioxidant status of glutathione peroxidase (GPx) and malondialdehyde (MDA) when compared to the control group. The serum level of IgM was significantly ( P<0.05 ) increased in group G3 and group G2 when compared to the control group. There were significant ( P<0.05 ) increases in total return from chick sales / LE (livre égyptienne) and net profit (one chick) / LE and in the economic efficiency of group G3 and group G2 when compared to the control group. Broiler chickens fed the diet supplemented with NUQO©NEX showed a positive effect in terms of the FCR, cholesterol, antioxidant status (GPx and MDA) and immune status (IgM), carcass quality traits, and economic effectiveness measurements.

## 1 Introduction

Poultry meat occupies an important position in our diet from a nutritional point of view (Marangoni et al., 2015; Prates, 2025). Poultry meat provides first-class protein as it includes every necessary amino acid in balanced proportion, necessary for maintaining life and promoting growth (Alagawany et al., 2021b). It is low in calories and is a good source of several essential fatty acids, as well as unsaturated and saturated fatty acids. Compared to other varieties of red meat, it has a higher protein level. Certain essential fatty acids like linoleic, linolenic, and arachidonic are present in chicken meat (Alagawany et al., 2021a; Kralik et al., 2025). Poultry meat is also an excellent source of vitamin B12 and niacin. Also, it has a relatively reliable source of riboflavin and thiamine. Poultry meat present in the diet contributes to various minerals (calcium, iron, and zinc) (Murphy and Allen, 2003). So poultry meat is a perfect food for infants, young children, adults, old people, those recovering from illness, and those trying to manage their weightiness (Kralik et al., 2025).

Many years ago, growth promoters of antibiotics (GPAs) were supplemented into animal and poultry feed (Reda et al., 2025). Risky or prolonged utilization of antibiotics causes numerous health problems for animals, as well as for humans. According to Stanaćev et al. (2011), the use of GPAs in the animal industry may lead to an increase in bacteria that are resistant to antibiotics, the buildup of antibiotic remains in animal products, and the potential for resistant strains to be passed from animals to humans through the food chain. Restrictions set by European Union health authorities in 2006 aimed to overcome this issue by limiting or outlawing the use of GPAs in egg- and meat-producing animals (Windisch et al., 2008). Any restriction on the use of GPAs in egg and meat production will raise the cost of production, therefore lowering the profitability. Following a complete ban or limited use of antibiotic growth promoters, thorough research about their replacement with chemicals that, with acceptable use, boost production performance, preserve animal health, and do not produce dangerous residues in food is necessary (Castanon, 2007; El-Ratel et al., 2024). Countries have therefore looked for natural substitutes that include antimicrobials in cattle and poultry diets without having a negative impact on health or productivity. In poultry production, research has demonstrated that antibiotic substitutes (prebiotics, enzymes, probiotics, emulsifiers, and organic acids) have some beneficial regulatory and antioxidant effects on intestinal microbiota. If the compounds' toxic and therapeutic effects are assessed in addition to their interactions with medications, they may be regarded as growth stimuli (Fatima et al., 2024). One of these important and promising alternatives is phytobiotics (Windisch et al., 2008). Thus, the hunt for plant extracts with therapeutic qualities was focused on adding them into the diet of birds as growth promoters (Alloui et al., 2013; Rafeeq et al., 2023).

Saponin (one of the phytogenic feed additives – PFAs) has the ability to enhance the permeability of the gut wall, hence potentially improving the absorption of minerals and nutrients (Reyer et al., 2017). In the meantime, some researchers have shown that feeding PFAs to broilers increased AME (apparent metabolizable energy) and crude protein digestibility (Murugesan et al., 2015; Mountzouris et al., 2011; Pirgozliev et al., 2019). As a result, PFAs will probably not increase the feed's digestible portion but would instead be used with the aim of maximizing the use of the non-digestible portion. Because of these advantages, a PFA can be included in the diet with nutritious matrix values, which is vital for feed sustainability and the lowest possible cost of formulation. On another hand, seaweeds are macroscopic organisms found in marine ecosystems and are a rich source of polyunsaturated fatty acids (PUFAs), polysaccharides, bioactive peptides, and enzymes (Matos et al., 2024). Seaweeds and their derivatives are important sources of animal and plant biostimulants and are frequently used to enhance agricultural productivity (Van Oosten et al., 2017). The most widely researched seaweed species, used as a source of biostimulants for commercial and industrial plants, is *Ascophyllum nodosum*. Many commercial products and extracts of *A. nodosum* have been shown to promote the growth of animals and plants and also to improve animal and plant defences by regulating physiological, molecular, and biochemical processes (Van Oosten et al., 2017).

For a long time, nutritional matrix values have been assigned to feed additives like enzymes (Saleh et al., 2023). This nutritional value is then included in the nutritional value of the entire diet, which permits the reduction of the incorporation of some ingredients, like those required for protein (soybean) and energy (oil and/or fat), as well as minerals (Bedford and Cowieson, 2020). This should theoretically result in a decrease in feed costs while the animal continues to perform at the same level of production. Hence, the purpose of this paper is to determine the effects of biostimulants as a natural growth promoter – on top and with a matrix value – in broiler diet on growth parameters and economic efficiency measurements.

## 2 Material and methods

### 2.1 Ethical approval

The study was conducted with ethical permission from Zagazig University and was approved by the Institutional Animal Care and Use Committee (approval no. ZU-IACUC/2/F/2/2025). We obtained informed consent from the owner of the birds involved in the study. All methods were carried out according to the relevant guidelines and regulations (Ethics Committee, Faculty of Agriculture, Zagazig University, Egypt). The study was carried out in compliance with the ARRIVE guidelines.

### 2.2 Feed additive used

NUQO©NEX is a blend of essential oils extracted from herbs and spices, including thyme, clove, and cinnamon, and *Ascophyllum nodosum*. NUQO©NEX is manufactured by NUQO (a French feed additives company) and is based on a specific component preparation and depends on a new double-encapsulation style, according to the manufacturer.

### 2.3 Experimental birds, housing, and management

Unsexed 1 d old chicks ( n=90 ) of a commercial meat type (Ross 308) purchased from a local hatchery were used as experimental birds in this study. On entrance, these chicks were weighed and randomly allocated to equal three treatment groups. Each group contained six replicates, with five chicks in each. Group G1 fed on a control diet (without additive), while groups G2 and G3 fed on a diet supplemented with 100 mg NUQO©NEX kg^−1^ diet (on top and matrix value, respectively, with the matrix value reducing the nutrient requirement by 0.3 % crude protein and 30 kcal metabolizable energy) for 35 d. Each experimental unit of the chicks was reared in a separate pen in a naturally ventilated open house at a density of 10 birds m^−2^. The birds were kept under similar managerial parameters, including space, temperature, light, relative humidity, and ventilation. The birds received continuous lighting during the first 5 d, and then the birds received a lighting regimen of 23 h of light and 1 h of darkness to minimize the chickens' activity. The temperature was set to 33 °C for the first week and then was regularly reduced by 3 °C per week and then was lastly kept at 24 °C.

Isocaloric and isonitrogenous diets were formulated to fulfil the nutritional requirements for broiler chicks as outlined in the Ross 308 Broiler Management Guide, which also specifies the dietary requirements for broilers weighing up to 2.0 kg. Fresh and clean water was available to the birds at all the times. Three mash rations (starter, grower, and finisher) were prepared and fed ad libitum to the experimental birds.

**Table 1:** Feed ingredients of experimental diets expressed on an as-fed basis (g kg^−1^).

	Starter			Grower			Finisher		
	G1	G2	G3	G1	G2	G3	G1	G2	G3
Corn	555.15	554.95	571.25	590.06	589.85	606.15	644.85	644.65	660.95
Soybean meal 48	363.51	363.54	353.61	328.44	328.47	318.55	276.27	276.30	266.37
Wheat bran	30.00	30.00	30.00	30.00	30.00	30.00	30.00	30.00	30.00
Soybean oil	8.99	9.06	2.10	14.25	14.32	7.36	13.14	13.21	6.25
Limestone	11.95	11.94	11.97	11.51	11.53	11.53	11.07	11.08	11.12
Mono-calcium phosphate	12.53	12.54	12.62	9.67	9.66	9.77	8.60	8.60	8.68
Sodium chloride	3.04	3.04	3.03	3.03	3.03	3.03	3.02	3.02	3.01
Sodium bicarbonate	1.51	1.51	1.52	1.52	1.52	1.53	1.55	1.55	1.56
L-methionine	3.74	3.74	3.80	3.21	3.21	3.26	3.00	3.00	3.05
L-lysine HCL	2.51	2.51	2.78	1.76	1.76	2.03	2.00	1.99	2.26
L-threonine	1.39	1.39	1.50	0.92	0.92	1.03	0.87	0.87	0.98
Choline chloride 60 %	0.88	0.88	0.92	0.83	0.83	0.86	0.83	0.83	0.87
Ronozyme proAct^a^	0.20	0.20	0.20	0.20	0.20	0.20	0.20	0.20	0.20
Ronozyme hi-phos^b^	0.10	0.10	0.10	0.10	0.10	0.10	0.10	0.10	0.10
Enspira^c^	0.10	0.10	0.10	0.10	0.10	0.10	0.10	0.10	0.10
Vitamin E	0.20	0.20	0.20	0.20	0.20	0.20	0.20	0.20	0.20
Broiler premix^e^	3.00	3.00	3.00	3.00	3.00	3.00	3.00	3.00	3.00
Mycofix select^d^	1.00	1.00	1.00	1.00	1.00	1.00	1.00	1.00	1.00
Diclazuril	0.20	0.20	0.20	0.20	0.20	0.20	0.20	0.20	0.20
NUQO©NEX	0.00	0.10	0.10	0.00	0.10	0.10	0.00	0.10	0.10
Total amount	1000	1000	1000	1000	1000	1000	1000	1000	1000

^a^ Ronozyme proAct (protease enzyme, DSM). ^b^ Ronozyme hi-phos (phytase enzyme, DSM). ^e^ Enspira (energy-releasing enzyme produced by United Animal Health). ^d^ Mycofix select (anti-mycotoxin, DSM). ^e^ Broiler premix in a 3 kg unit and contribution per kg of complete feed: vitamin A – 13 000 IU; vitamin D3 – 5000 IU; vitamin E – 80 mg; vitamin K3 – 4 mg; vitamin B1 – 5 mg; vitamin B2 – 9 mg; vitamin B6 – 5 mg; pantothenic acid – 25 mg; biotin – 0.350 mg; folic acid – 2.5 mg; niacin – 70 mg; vitamin B12 – 0.02 mg; I (calcium iodate) – 1.25 mg; Se (sodium selenite) – 0.300 mg; Cu (copper sulfate) – 16 mg; Mn (manganese sulfate) – 120 mg; Zn (zinc oxide) – 120 mg; Fe (iron sulfate) – 20 mg. ME denotes metabolic energy. CP denotes crude protein. EE denotes ether extract. CF denotes crude fibre.

**Table 2:** Composition of experimental diets expressed on an as-fed basis (g kg^−1^).

	Starter			Grower			Finisher		
	G1	G2	G3	G1	G2	G3	G1	G2	G3
Calculated analysis^∗^									
ME, kcal kg^−1^	2975	2975	2975	3050	3050	3050	3100	3100	3100
CP, %	23.00	23.00	23.00	21.50	21.50	21.50	19.50	19.50	19.50
EE, %	3.84	3.85	3.19	4.42	4.43	3.77	4.40	4.41	3.75
CF, %	3.07	3.07	3.07	3.00	3.00	2.99	2.89	2.89	2.88
Calcium, %	0.96	0.96	0.96	0.88	0.88	0.88	0.83	0.83	0.83
Available P, %	0.50	0.50	0.50	0.44	0.44	0.44	0.41	0.41	0.41
Lysine, %	1.32	1.32	1.32	1.18	1.18	1.18	1.08	1.08	1.08
Methionine, %	0.69	0.69	0.69	0.62	0.62	0.63	0.58	0.58	0.58

^∗^ ME denotes metabolic energy. CP denotes crude protein. EE denotes ether extract. CF denotes crude fibre.

The experiment was extended for 35 d, and the feeding period was divided into three phases: the starter phase from 0 to 10 d, in which the birds were fed a diet containing 23 % CP and ME (2975 kcal kg^−1^ diet), the grower phase from 11 to 24 d, in which the birds were fed a diet containing 21.5 % CP and ME (3050 kcal kg^−1^ diet), and the finisher phase from 25 to 35 d, in which the birds were fed a diet containing 19.5 % CP and ME (3100 kcal kg^−1^ diet) (Tables 1 and 2). The experimental diets and feedstuffs were examined for ether extract, moisture, and crude protein in accordance with the AOAC's (2002) standard operating procedures.

### 2.4 Indicators for assessing growth performance

On their first day of life, each bird was independently weighed to ascertain the average beginning body weight; then, the body weight was noted every week to estimate the average body weight development of the birds in every group. Each group's weekly body weight gain was computed for each bird each day, and the average weight gain per bird and per group was then determined as follows: body weight gain = W2 - W1. Regarding feed intake (FI), every day, the diets were routinely delivered at 03:00 p.m. (LT); daily feed consumption per group was computed by subtracting the weight of the remaining feed from the weight of the given feed and subsequently dividing by the number of birds in each group. The feed conversion ratio (FCR) was estimated weekly according to Wagner et al. (1983). The European production efficiency factor (EPEF) was estimated following Huff et al. (2013) as follows: EPEF = viability (%) ⋅ body weight (kg) ⋅ 100 / feed conversion ratio ⋅ trial duration in days.

### 2.5 Carcass traits

When the 35 d trial ended, four birds from each group were chosen, fasted for the entire night, were weighed, and then were slaughtered with a sharp knife and drained until all of the blood was gone. The feathers were then removed, the animals were disembowelled, and the weight was finally taken to determine the dressing percentage. The liver, heart, digestive tract (gizzard, proventriculus, and spleen) were selected, weighed, and expressed as a percentage of live body weight (Dilger et al., 2006).

### 2.6 Biochemical analyses of the serum

#### 2.6.1 Blood sampling

Four birds from each group were randomly selected at the end of the study (35 d of age) and were slaughtered to obtain blood samples. Each blood sample was collected in a clean centrifuge tube without anticoagulant to separate the serum. The samples were refrigerated overnight and then centrifuged at 3000 rpm for 15 min. Before being used for biochemical analysis, the serum was stored at -20 °C.

#### 2.6.2 Serum parameters measured

Serum total cholesterol (Natio and Kaplan, 1984), triglyceride, high-density lipoprotein level (Burstein and Scholnick, 1973), low-density lipoprotein level (Friedewald et al., 1972), total protein (Grant, 1987), albumin (Doumas and Pinkas, 1971), globulin (Doumas and Biggs, 1972), creatinine, and blood urea nitrogen (Fossati et al., 1980) were calorimetrically determined using commercial diagnostic kits. As explained by Reitman and Frankel (1957), the serum levels of the enzymes aspartate-aminotransferase (AST) and alanine-aminotransferase (ALT) were measured. Glutathione peroxidase (GPx) activity was measured calorimetrically in erythrocytes following the procedures of Rotruck et al. (1973), and serum malondialdehyde (MDA) concentration was measured in accordance with Yagi (1984) using a spectrophotometer at 520 nm and being expressed as n mal mL^−1^ in terms of the TBARS (thionbarbituric reaction substance) index. Following manufacturer directions, ELISA tests (Bird IgY/IgM ELISA Quantitation kits; Shanghai Enzyme-linked Biotechnology Co. Ltd., Shanghai, China) were used to determine the quantities of serum immunoglobulins, including immunoglobulin Y (IgY) and immunoglobulin M (IgM).

### 2.7 Economic efficiency measurements

Cost parameters were categorized using the techniques of Ahmed (2007). The first parameter was total fixed costs (TFCs): in this state, each chick took up equal costs in terms of litter, labour, purchased chicks, water, electrolyte, veterinary medicaments (drugs, vaccine and veterinary supervision), equipment depreciation, and building, and so these factors were considered to be fixed expenses for each group. Next were the total variable costs (TVCs), covering the expense of feed and feed additives (Hassan, 2009). This was calculated during the experiment. Total costs (TCs) were calculated when the entirety of the fixed expenses and variable costs were added up. Total returns (TRs) from chick sales were calculated as body weight × kg price. Net profit was calculated as total returns - total costs. Economic efficiency was calculated as net profit / total cost.

**Table 3:** Effect of dietary supplementation of NUQO©NEX on growth performance parameters of broiler chickens during the experimental period (1–35 d of age).

	Treatments^1^			p value
Parameter	G1	G2	G3	
Initial body weight (g)	44.3±0.17	44.42±0.22	44.2±0.40	0.867
Final body weight (g)	2192±15.27	2223±19.45	2216±19.34	0.486
Absolute body weight gain (g)	2147±15.39	2178±19.57	2172±19.73	0.494
Total feed intake (g per bird)	3369±22.13	3301±26.28	3356±33.05	0.255
Feed conversion ratio (g feed / g gain)	1.57±0.005 ^a^	1.52±0.003 ^c^	1.55±0.003 ^b^	<0.001
EPEE^2^	365±16.34	397±17.57	388±12.34	0.384

^a–c^ Means within the rows carrying different superscripts are significantly different at p<0.05 . ^1^ Treatments: G1 – control diet (no additives added); G2 – control diet +100 mg NUQO©NEX (biostimulants) kg^−1^ diet (on top); G3: control diet +100 mg NUQO©NEX (biostimulants) kg^−1^ diet (matrix value reducing nutrient requirement by 0.3 % crude protein and 30 Kcal metabolizable energy). ^2^ EPEF: European production efficiency factor.

### 2.8 Statistical analyses

A completely statistical randomization design was employed in this investigation. The conventional statistical one-way analysis of variance (ANOVA) was applied to all outcomes using SPSS (2008), version 17. Duncan's multiple-range test was used to determine whether or not the means of the treatments differed significantly (Duncan, 1955). Means were considered to be statistically significant at p≤0.05 .

## 3 Results

### 3.1 Growth performance parameters

The effect of the dietary supplementation of NUQO©NEX on the overall performance of broiler chicks during the starter, grower, and finisher periods is shown in Table 3. Statistical analysis of the data showed that experimental diets had a significant ( P<0.05 ) effect on the FCR of broiler chicks during the experiment. Broiler chickens from groups G2 and G3 showed a significant improvement ( P<0.05 ) in terms of the average feed conversion ratio compared to the control group. G2 recorded the best value of FCR compared to group G3 and G1. No significant ( P>0.05 ) difference was found in final body weight (g), absolute body weight gain (g), total feed intake (g per bird), and EPEF when groups G2 and G3 were compared to the control group. However, there was a slight improvement in the EPEF in the groups G2 and G3 when compared to the control group.

**Table 4:** Effect of dietary supplementation of NUQO©NEX on the lipid profile of broiler chickens at 35 d of age.

Parameter^2^	Treatments^1^			p value
	G1	G2	G3	
Cholesterol (mg dL^−1^)	210±17.25 ^a^	167±6.66 ^b^	178±7.17 ^ab^	0.036
TG (mg dL^−1^)	164±7.94	148±6.49	154±3.24	0.190
HDL (mg dL^−1^)	59.25±1.59	55.67±3.12	60.42±2.67	0.400
LDL (mg dL^−1^)	111±15.93 ^a^	82.47±6.56 ^b^	87.71±5.67 ^b^	0.029
VLDL (mg dL^−1^)	32.87±1.58	29.62±1.29	30.83±0.65	0.190

^ab^ Means within the rows carrying different superscripts are significantly different at P<0.05 . ^1^ Treatments: G1 – control diet (no additives added); G2 – control diet + 100 mg NUQO©NEX (biostimulants) kg^−1^ diet (on top); G3 – control diet + 100 mg NUQO©NEX (biostimulants) kg^−1^ diet (matrix value reducing nutrient requirement by 0.3 % crude protein and 30 Kcal metabolizable energy). ^2^ TG: triglyceride, HDL: high-density lipoprotein, LDL: low-density lipoprotein, VLDL: very-low-density lipoprotein

### 3.2 Serum parameters

#### 3.2.1 Lipid profile

The effect of the dietary supplementation of NUQO©NEX on the lipid profile of broiler chicks during the experimental period is shown in Table 4. Cholesterol and LDL levels were significantly ( P<0.05 ) decreased in group G2 and then in group G3 of the broiler chicks when compared to the control group. In contrast, no significant difference was detected in the TG, HDL, and VLDL among the different experimental groups.

**Table 5:** Effect of dietary supplementation of NUQO©NEX on the liver and kidney functions of broiler chickens at 35 d of age.

Parameter^b^	Treatments^a^			p value
	G1	G2	G3	
Total protein (g dL^−1^)	6.63±0.43	6.09±0.38	6.04±0.38	0.525
Albumin (g dL^−1^)	2.23±0.06	2.15±0.05	2.24±0.16	0.826
Globulin (g dL^−1^)	4.40±0.41	3.94±0.41	3.80±0.37	0.544
AST (IU L^−1^)^a^	228±5.98	198±7.45	213±13.54	0.094
ALT (IU L^−1^)^a^	9.70±1.09	9.62±0.72	9.42±0.78	0.972
Urea (mg dL^−1^)	14.67±0.70	15.06±0.32	15.12±0.44	0.798
Creatinine (mg dL^−1^)	0.42±0.03	0.41±0.01	0.36±0.02	0.260

Means within the rows carrying different superscripts are significantly different at P<0.05 . ^a^ Treatments: G1 – control diet (no additives added); G2 – control diet + 100 mg NUQO©NEX (biostimulants) kg^−1^ diet (on top); G3: control diet + 100 mg NUQO©NEX (biostimulants) kg^−1^ diet (matrix value reducing nutrient requirement by 0.3 % crude protein and 30 Kcal metabolizable energy). ^b^ AST: aspartate-aminotransferase, ALT: alanine-aminotransferase

#### 3.2.2 Liver and kidney functions

Table 5 shows the effect of the dietary supplementation of NUQO©NEX on the liver function of broiler chicks during the experimental period. The serum of the broiler chickens of the experimental groups showed no significant ( P>0.05 ) difference in terms of total protein, albumin, globulin, AST, and ALT among the different the experimental groups. In the same context, the data presented in Table 5 showed no significant difference in terms of all kidney parameters among treatments.

**Table 6:** Effect of dietary supplementation of NUQO©NEX on antioxidant indices of broiler chickens at 35 d of age.

Parameter^2^	Treatments^1^			p value
	G1	G2	G3	
GPx (ng mL^−1^)	1.58±0.01 ^c^	2.58±0.02 ^b^	2.67±0.02 ^a^	<0.001
MDA (nmol mL^−1^)	0.94±0.01 ^a^	0.69±0.01 ^b^	0.59±0.01 ^c^	<0.001

^a–c^ Means within the rows carrying different superscripts are significantly different at P<0.05 . ^1^ Treatments: G1 – control diet (no additives added); G2 – control diet + 100 mg NUQO©NEX (biostimulants) kg^−1^ diet (on top); G3 – control diet + 100 mg NUQO©NEX (biostimulants) kg^−1^ diet (matrix value reducing nutrient requirement by 0.3 % crude protein and 30 Kcal metabolizable energy). ^2^ GPx: glutathione peroxidase, MDA: Gaweł et al. (2004).

#### 3.2.3 Antioxidant activity

The effect of the dietary supplementation of NUQO©NEX on the antioxidant activity of broiler chicks during the experiment is shown in Table 6. The serum of the broiler chickens of the experimental groups G2 and G3 showed a significant ( P<0.05 ) increase in the antioxidant status of GPx when compared to the control group. The highest value of GPx was found in the group with 100 mg NUQO©NEX kg^−1^ diet (on matrix). Statistical analysis of the data showed that serum MDA level was significantly decreased ( P<0.05 ) in group G3 and group G2 when compared to the control group. The lowest value of MDA concentration was found in group G3.

**Table 7:** The effect of dietary supplementation of NUQO©NEX on the immunoglobulins of broiler chickens at 35 d of age.

Parameter^2^	Treatments^1^			p value
	G1	G2	G3	
IgY (mg dL^−1^)	2.67±0.19	3.14±0.19	3.28±0.20	0.074
IgM (mg dL^−1^)	1.78±0.11 ^b^	2.36±0.20 ^a^	2.44±0.20 ^a^	0.026

^a,b^ Means within the rows carrying different superscripts are significantly different at P<0.05 . ^1^ Treatments: G1 – control diet (no additives added); G2 – control diet + 100 mg NUQO©NEX (biostimulants) kg^−1^ diet (on top); G3 – control diet + 100 mg NUQO©NEX (biostimulants) kg^−1^ diet (matrix value reducing requirement reduced by 0.3 % crude protein and 30 Kcal metabolizable energy). ^2^ IgY and IgM: immunoglobulins

#### 3.2.4 Immunoglobulins

The results shown in Table 7 showed that the serum IgM level was significantly increased ( P<0.05 ) in group G3 and group G2 when compared to G1. The highest value of IgM level was found in group G3. In contrast, no significant difference was detected in the IgY level among the different experimental groups.

**Table 8:** Effect of dietary supplementation of NUQO©NEX on carcass traits relative to the live weight of broiler chickens at 35 d of age.

Parameter^2^	Treatments^1^			p value	
	G1	G2	G3		
Live weight per bird g^−1^	2192±13.95	2223±10.55	2224±10.64	0.101	
Carcass yield per bird g^−1^	1588±13.31 ^b^	1638±10.46 ^a^	1655±12.38 ^a^	0.001	
Heart %	0.50±0.005 ^c^	0.52±0.005 ^b^	0.56±0.006 ^a^	<0.001	
Liver %	2.07±0.02 ^c^	2.31±0.01 ^b^	2.37±0.01 ^a^	<0.001	
Gizzard %	1.72±0.01 ^c^	2.08±0.01 ^b^	2.20±0.02 ^a^	<0.001	
Proventriculus %	0.31±0.007 ^c^	0.36±0.006 ^a^	0.34±0.007 ^b^	<0.001	
Spleen %	0.10±0.003 ^c^	0.11±0.002 ^b^	0.12±0.001 ^a^	<0.001	
Dressing %	77.04±0.19 ^c^	78.96±0.18 ^b^	79.86±0.24 ^a^	<0.001	
Giblets %	4.60±0.02 ^c^	5.27±0.03 ^b^	5.47±0.04 ^a^	<0.001	
Carcass yield %	72.44±0.18 ^c^	73.69±0.15 ^b^	74.39±0.22 ^a^	<0.001	

^a–c^ Means within the rows carrying different superscripts are significantly different at P<0.05 . ^1^ Treatments: G1 – control diet (no additives added); G2 – control diet + 100 mg NUQO©NEX (biostimulants) kg^−1^ diet (on top); G3 – control diet + 100 mg NUQO©NEX (biostimulants) kg^−1^ diet (matrix value reducing requirement by 0.3 % crude protein and 30 Kcal metabolizable energy).

### 3.3 Carcass traits

The data presented in Table 8 illustrate the effect of feeding broiler chickens diets with different levels of NUQO©NEX on carcass traits. The carcass yield per bird per gram, dressing percentage, and carcass yield percentage of broiler chickens were significantly increased ( P<0.05 ) in group G3 and group G2 when compared to the control group. When compared to the control group, the live weight per bird per gram had no significant ( P>0.05 ) effect on group G3 and group G2. The data showed that there were significant ( P<0.05 ) increases in the percentage of heart, liver, gizzard, proventriculus, spleen, and giblets of group G3 and group G2 when compared to the control group.

**Table 9:** Effect of dietary supplementation of NUQO©NEX on economic evaluation of broiler chickens during the experimental period (1–35 d of age).

Parameter^2^	Treatments^1^			p value
	G1	G2	G3	
Feed cost (one chick) / LE	90.29±0.58	88.79±0.69	88.71±0.86	0.958
Total cost / LE	110.29±0.58	108.79±0.69	108.71±0.86	0.958
Total returns / LE	131.53±0.91 ^b^	133.39±1.16 ^a^	133.01±1.16 ^a^	0.038
Net profit (one chick) / LE	21.24±0.44 ^b^	24.6±0.51 ^a^	24.3±0.37 ^a^	<0.001
Economic efficiency	0.19±0.004 ^b^	0.23±0.004 ^a^	0.22±0.003 ^a^	<0.001

^a,b^ Means within the rows carrying different superscripts are significantly different at P<0.05 . ^1^ Treatments: G1 – control diet (no additives added); G2 – control diet + 100 mg NUQO©NEX (biostimulants) kg^−1^ diet (on top); G3 – control diet + 100 mg NUQO©NEX (biostimulants) kg^−1^ diet (matrix value reducing nutrient requirement by 0.3 % crude protein and 30 Kcal metabolizable energy).

### 3.4 Economic efficiency measurements

Table 9 shows the effect of NUQO©NEX on economic efficiency. There was no significant ( P>0.05 ) difference in the feed cost (one chick) / LE and total cost / LE of group G3 and group G2 when compared to the control group. The use of NUQO©NEX in the birds' feeding systems improved the total return from chick sales / LE, net profit (one chick) / LE, and economic efficiency when compared to the control group.

## 4 Discussion

Statistical analysis of the current data showed that the broiler chicken diet supplemented with NUQO©NEX exhibited a significant improvement ( P<0.05 ) in FCR compared to the control group. The use of the 100 mg NUQO©NEX kg^−1^ diet (on top) achieved the best significant FCR compared to the 100 mg NUQO©NEX kg^−1^ diet (with matrix) and the control group. No significant ( P>0.05 ) difference was found in the final body weight (g), absolute body weight gain (g), total feed intake (g per bird), and EPEF when groups were supplemented with NUQO©NEX when compared to to control group. However, there was a slight improvement in the EPEF in the groups supplemented with NUQO©NEX when compared to control group. According to Platel and Srinivasan (2004), the main reason for the improvement in gut health is believed to be the possible stimulatory activity of phytogenic chemicals on digestive secretions like bile, mucus, and digestive enzymes. Our results agree with those of Amad et al. (2011), who proved that the FCR of broiler chickens fed PFA was higher. This may be connected to the fact that minerals, fats, and proteins have greater nutrient digestibility. These consequences may lead to increased economic efficiency in the production of meat from broilers.

In a study conducted by Cho et al. (2014), adding 250 mg kg^−1^ of anise and thyme to broiler diets as feed supplements significantly increased the feed conversion rate. Also, Bravo et al. (2011) found that the FI in broilers supplemented with PFA (i.e. 100 mg kg^−1^) was unaffected by a 2 % reduction in metabolizable-energy (ME) level (i.e. from 3000 to 2950 kcal kg^−1^) while maintaining a consistent crude protein (CP) level. In line with Paraskeuas et al. (2016), broiler growth performance was unaffected by PFA addition, but FCR was raised, resulting in reductions in the formulated diet of 1.32 % crude protein and ME by 0.8 MJ kg^−1^. Similarly to this, leaf meal from medicinal plants increased the FCR and growth of broilers by enhancing their antioxidative ability without having an adverse impact on their health (Daramola, 2019). Kleczek et al. (2014), in a study looking into the impact of propolis on broiler growth performance, similarly discovered that there were no significant differences in EPEF values.

According to studies by Attia et al. (2017a, b) and Jamroz et al. (2005), adding essential oil (EO) supplements to the diet enhanced the growth performance of the chicks by promoting the release of digestive enzymes, which, in turn, leads to better nutrient digestion. Statistical analysis of the lipid profile of the present study showed that cholesterol and LDL levels were significantly decreased ( P<0.05 ) in the group supplemented with the 100 mg NUQO©NEX kg^−1^ diet (with matrix) and then in the group supplemented with the 100 mg NUQO©NEX kg^−1^ diet (on top).

In contrast, no significant difference was detected in TG, HDL, and VLDL among the different experimental groups. Numerous herbal spices or extracts that were studied, including the essential oils of oregano (Brenes and Roura, 2010) and thyme (Lee et al., 2005), demonstrated good hypo-cholesterolemic effects in chickens. These outcomes could primarily be produced by the enzymes involved in the suppression of lipid and cholesterol production (Crowell, 1999). Also, broiler chickens given diets supplemented with carvacrol, thymol, and menthol showed a drop in serum cholesterol, but there were no alterations observed in the blood serum concentrations or ratios of triglycerides, HDL, and LDL (Saadat et al., 2016; Beiranvand et al., 2017). Our results are consistent with those of Abdel-Wahab (2019), who reported that, when feeding broiler chickens with diets containing marjoram plant, which contains thymol and carvacrol, their serum levels of LDL-c, cholesterol, and triglycerides dropped substantially, while their levels of HDL-c rose.

Statistical analysis of the liver and kidney functions of the present data showed that the serum of broiler chickens showed no significant ( P>0.05 ) difference in total protein, albumin, globulin, AST, ALT urea, or creatinine among the different experimental groups. Our results agree with those of Khosravinia et al. (2013), who reported that, when birds drink water that contains a kind of SKEO (essential oil), there is no rise in their levels of creatinine or uric acid. Mansoub (2011) found that blood biochemical measures of the liver and kidney functions were not significantly affected by adding oregano oil. On the other hand, Abdel-Wahab (2019) found that adding marjoram to broiler diets substantially lowered liver enzymes (ALT and AST).

Abou-Elkhair et al. (2014) found that supplementing broiler chick diets with black pepper increased their blood total protein and albumin levels. Statistical analysis of the present data showed that the broiler diets supplemented with NUQO©NEX recorded significant ( P<0.05 ) increases in the antioxidant status of GPx when compared to the control group. The highest value of GPx concentration was found in the group supplemented with the 100 mg NUQO©NEX kg^−1^ diet (with matrix).

In another study, Mehdipour and Afsharmanesh (2018) stated that, on day 42 of the trial, dietary cinnamon powder markedly raised total antioxidant capacity (TAC), total SOD activity, corticosteroid, and catalase in the group fed cinnamon, and there was a decrease in the levels of MDA. Statistical analysis of the present data showed that MDA level was decreased ( P<0.05 ) in groups supplemented with NUQO©NEX when compared to the control group. The lowest value of MDA concentration was found in group supplemented with the 100 mg NUQO©NEX kg^−1^ diet (with matrix).

Statistical analysis of the data showed that the IgM level was significantly increased ( P<0.05 ) in groups supplemented with NUQO©NEX when compared to the control group. The highest value of IgM level was found in the group supplemented with the 100 mg NUQO©NEX kg^−1^ diet (with matrix). In contrast, no significant difference was detected in IgY levels among different experimental groups. Similarly to our findings, following a commercial blend of phytogenic supplementation, Hong et al. (2012) found that IgG and total antibody titres were unaffected by essential oil supplementation (125 ppm including essential oil derived from oregano, anise, and citrus peel). In addition, Sang-Oh et al. (2013) reported that supplementing cinnamon in the diet raised the levels of immunoglobulin in the serum of broilers. Furthermore, certain serum biochemical data indicate that the use of biostimulants can enhance immunological function. Begum et al. (2014) stated that the serum immunoglobulin (Ig) G concentration rose when 0.1 % of a blend of herbal extracts was added to broiler diets.

The carcass yield per bird per gram, dressing percentage, and carcass yield percentage of broiler chickens were significantly increased ( P<0.05 ) in groups supplemented with NUQO©NEX when compared to the control group. The live weight per bird per gram had no significant ( P>0.05 ) effect on groups supplemented with NUQO©NEX when compared to control group. Statistical analysis of the present data showed that there were significant ( P<0.05 ) increases in the percentage of heart, liver, gizzard, proventriculus, spleen, and giblets of the groups supplemented with NUQO©NEX when compared to control group. Our results are consistent with those of Abo Omar et al. (2016), who reported that, when natural herbs were added to the feed, the proportions of dressings were greater than those found in the control birds.

In the present study, there was no significant ( P>0.05 ) difference in the feed cost (one chick) / LE and total cost / LE of groups supplemented with NUQO©NEX when compared to the control group. Statistical analysis of the present data showed that there were significant ( P<0.05 ) increases in the total returns from chick sales / LE, net profit (one chick) / LE, and economic efficiency of groups supplemented with NUQO©NEX when compared to the control group. Yan et al. (2019) mentioned that adding phytonutrient supplements to a low-energy diet helps to keep broiler FCR high and to boost economic profit, possibly through better gut health.

## 5 Conclusions

Broiler chickens fed a diet supplemented with (NUQO©NEX) showed positive effects on FCR, cholesterol, LDL, antioxidant status (GPx and MDA) and immune state (IgM), carcass quality traits, and economic efficiency measurements (total return from chick sales / LE and net profit (one chick)). The best results recorded were with the inclusion rate of 100 mg NUQO©NEX kg^−1^ (on top).

## Data Availability

All the data generated or analysed during this study are included in this published article.

## References

[bib1.bib1] Abdel-Wahab AA (2019). Effect of adding Marjoram powder to broiler chicks diet on performance, blood and antioxidant enzyme activity. Egypt J Nutr Feeds.

[bib1.bib2] Abo Omar J, Hejazi A, Badran R (2016). Performance of broilers supplemented with natural herb extract. Open J Anim Sci.

[bib1.bib3] Abou-Elkhair R, Ahmed H, Selim S (2014). Effects of black pepper (*Piper Nigrum*), turmeric Powder (*Curcuma Longa*) and Coriander Seeds (*Coriandrum Sativum*) and their combinations as feed additives on growth performance, carcass traits, some blood parameters and humoral immune. Asian-Australas J Anim Sci.

[bib1.bib4] Ahmed IAM (2007). Economic and productive efficiency of poultry farms in relation to veterinary management.

[bib1.bib5] Alagawany M, Elnesr SS, Farag MR, ElSabrout K, Alqaisi O, Dawood MAO, Soomro H, Abdelnour SA (2021). Nutritional significance and health benefits of omega-3, -6 and -9 fatty acids in animals. Anim Biotechnol.

[bib1.bib6] Alagawany M, Elnesr SS, Farag MR, Tiwari R, Yatoo MI, Karthik K, Michalak I, Dhama K (2021). Nutritional significance of amino acids, vitamins and minerals as nutraceuticals in poultry production and health-A comprehensive review. Vet Quar.

[bib1.bib7] Alloui MN, Szczurek W, Świątkiewicz S (2013). The usefulness of prebiotics and probiotics in modern poultry nutrition. Ann Anim Sci.

[bib1.bib8] Amad AA, Männer K, Wendler KR, Neumann K, Zentek J (2011). Effects of a phytogenic feed additive on growth performance and ileal nutrient digestibility in broiler chickens. Poult Sci.

[bib1.bib9] Association of Official Analytical Chemists (AOAC) (2002). Official Methods of Analysis.

[bib1.bib10] Attia YA, Al-Harthi MA, Hassan SS (2017). Turmeric (*Curcuma longa* Linn.) as a phytogenic growth promoter alternative for antibiotic and comparable to mannan oligosaccharides for broiler chicks. Rev Mexicana Ciencias Pecuarias.

[bib1.bib11] Attia YA, Bakhashwain AA, Bertu NK (2017). Thyme oil (Thyme vulgaris L.) as a natural growth promoter for broiler chickens reared under hot climate. Ital J Anim Sci.

[bib1.bib12] Bedford MR, Cowieson AJ (2020). Matrix values for exogenous enzymes and their application in the real world. J Appl Poult Res.

[bib1.bib13] Begum M, Hosain MM, Kim IH (2014). Effects of the plant extract YGF251 on growth performance, meat quality, relative organ weight, nutrient digestibility and blood profiles in broiler chickens: possible role of insulin-like growth factor 1. Vet Medicina.

[bib1.bib14] Beiranvand M, Amin M, Shahraki AH, Romani B (2017). Antimicrobial activity of endophytic bacterial populations isolated from medical plants of Iran. Ir J Microbiol.

[bib1.bib15] Bravo D, Utterback P, Parsons CM (2011). Evaluation of a mixture of carvacrol, cinnamaldehyde and capsicum oleoresin for improving growth performance and metabolizable energy in broiler chicks fed corn and soybean meal. J Appl Poult Res.

[bib1.bib16] Brenes A, Roura E (2010). Essential oils in poultry nutrition: main Effects and modes of action. Anim Feed Sci Technol.

[bib1.bib17] Burstein M, Scholnick HR (1973). Lipoprotein polyanion-metal interaction. Adv Lipid Res.

[bib1.bib18] Castanon JI (2007). History of the use of antibiotics as growth promoters in European poultry feeds. Poult Sci.

[bib1.bib19] Cho JH, Kim HJ, Kim IH (2014). Effects of phytogenic feed additive on growth performance, digestibility, blood metabolites, intestinal microbiota, meat color and relative organ weight after oral challenge with *Clostridium perfringens* in broilers. Livest Sci.

[bib1.bib20] Crowell PL (1999). Prevention and therapy of cancer by dietary monoterpenes. J Nutr.

[bib1.bib21] Daramola OT (2019). Medicinal plant leaf meal supplementation in broiler chicken's diets: effect on performance characteristics, serum metabolite and antioxidant status. Anim Res Int.

[bib1.bib22] Dilger RN, Martinez Amezcua C, Pillai PB, Emmert JL, Parsons CM, Baker DH (2006). Effect of Reciprocating dietary lysine fluctuations on chick growth and carcass yield. Poult Sci.

[bib1.bib23] Doumas BT, Biggs HG (1972). Standard methods of clinical chemistry.

[bib1.bib24] Doumas BT, Pinkas M (1971). Albumin standards and the measurement of serum albumin with bromo cresol green. Clin Chem Acta.

[bib1.bib25] Duncan DB (1955). Multiple Range and Multiple F-Tests. Biometrics.

[bib1.bib26] El-Ratel IT, EL-Deep MH, Alharbi NK, Alyoubi WAA, El-Kholy KH, Badawy AA, Ahmed AE, El Basuini MFM, Alagawany M, Fouda SF (2024). Lysozyme as an alternative to antibiotics improves growth, antioxidants status, immunity, and intestinal bacteria in broiler chickens during the fattening period. Arch Anim Breed.

[bib1.bib27] Fatima FT, Asad M, Qadeer S, Hira T, Fatima A, Anjum T, Zafar A, Shaheen K, Bibi T, Parveen S, Abbas RZ, Akhtar T, Asrar R, Khan AMA, Saeed Z (2024). Complementary and alternative medicine: feed additives.

[bib1.bib28] Fossati P, Prencipe L, Berti G (1980). Enzymatic colorimetric method of determination of uric acid in serum. Clinical Chemistry.

[bib1.bib29] Friedewald WT, Levy RI, Fredrickson DS (1972). Estimation of the concentration of low-density lipoprotein cholesterol in plasma, without use of the preparative ultracentrifuge. Clin Chem.

[bib1.bib30] Gaweł S, Wardas M, Niedworok E, Wardas P (2004). Dialdehyd malonowy (MDA) jako wskaźnik procesów peroksydacji lipidów w organizmie [Malondialdehyde (MDA) as a lipid peroxidation marker]. Wiad Lek.

[bib1.bib31] Grant GH, Tietz NW (1987). Fundamentals of Clinical Chemistry.

[bib1.bib32] Hassan FA (2009). Effect of some feed additives on economic and productive efficiency of broilers [master's thesis].

[bib1.bib33] Hong JC, Steiner T, Aufy A, Lien TF (2012). Effects of supplemental essential oil on growth performance, lipid metabolites and immunity, intestinal characteristics, microbiota and carcass traits in broilers. Livest Sci.

[bib1.bib34] Huff GR, Huff WE, Jalukar S, Oppy J, Rath NC, Packialakshmi B (2013). The effects of yeast feed supplementation on turkey performance and pathogen colonization in a transport stress/Escherichia coli challenge. Poult Sci.

[bib1.bib35] Jamroz D, Wiliczkiewicz A, Werteleck T, Orda J, Sukorupinska J (2005). Use of active substances of plant origin in chicken diets based on maize and locally grown cereals. Br Poult Sci.

[bib1.bib36] Khosravinia H, Ghasemi S, Alavi ER (2013). The effect of savory (*Satureja khuzistanica*) essential oils on performance, liver and kidney functions in broiler chickens. J Anim Feed Sci.

[bib1.bib37] Kleczek K, Wilkiewicz-Wawro E, Wawro K, Makowski W, Murawska D, Wawro M (2014). The effect of dietary propolis supplementation on the growth performance of broiler chickens. Pol J Nat Sci.

[bib1.bib38] Kralik Z, Kralik G, Košević M (2025). Effects of vegetable oils supplemented into broiler diet on the fatty acid profile and lipid indices in broiler meat. Agriculture.

[bib1.bib39] Lee SJ, Umano K, Shibamoto T, Lee KG (2005). Identification of volatile components in basil (*Ocimum basilicum* L.) and thyme leaves (*Thymus vulgaris* L.) and their antioxidant properties. Food Chem.

[bib1.bib40] Mansoub NH (2011). Assessment on effect of thyme on egg quality and blood parameters of laying hens. Annal Biol Res.

[bib1.bib41] Marangoni F, Corsello G, Cricelli C, Ferrara N, Ghiselli A, Lucchin L, Poli A (2015). Role of poultry meat in a balanced diet aimed at maintaining health and wellbeing: an Italian consensus document. Food Nutr Res.

[bib1.bib42] Matos J, Cardoso C, Serralheiro ML, Bandarra NM, Afonso C (2024). Seaweed bioactives potential as nutraceuticals and functional ingredients: A review. J Food Comp Anal.

[bib1.bib43] Mehdipour Z, Afsharmanesh M (2018). Evaluation of synbiotic and cinnamon (*Cinnamomum verum*) as antibiotic growth promoter substitutions on growth performance, intestinal microbial populations and blood parameters in Japanese quail. J Livest Sci Technol.

[bib1.bib44] Mountzouris KC, Paraskevas V, Tsirtsikos P, Palamidi I, Steiner T, Schatzmayr G, Fegeros K (2011). Assessment of a phytogenic feed additive effect on broiler growth performance, nutrient digestibility and caecal microflora composition. Anim Feed Sci Technol.

[bib1.bib45] Murphy SP, Allen LH (2003). Nutritional importance of animal source foods. The Journal of Nutrition.

[bib1.bib46] Murugesan GR, Syed B, Haldar S, Pender C (2015). Phytogenic feed additives as an alternative to antibiotic growth promoters in broiler chickens. Front Vet Sci.

[bib1.bib47] Natio H, Kaplan A (1984). Cholesterol. Clinical Chemistry.

[bib1.bib48] Paraskeuas V, Fegeros K, Palamidi I, Theodoropoulos G, Mountzouris KC (2016). Phytogenic administration and reduction of dietary energy and protein levels affects growth performance, nutrient digestibility and antioxidant status of broilers. J Poult Sci.

[bib1.bib49] Pirgozliev V, Mansbridge SC, Rose SP, Lillehoj HS, Bravo D (2019). Immune modulation, growth performance, and nutrient retention in broiler chickens fed a blend of phytogenic feed additives. Poult Sci.

[bib1.bib50] Platel K, Srinivasan K (2004). Digestive stimulant action of spices: A myth or reality?. Indian J Med Res.

[bib1.bib51] Prates JAM (2025). Nutritional value and health implications of meat from monogastric animals exposed to heat stress. Nutrients.

[bib1.bib52] Rafeeq M, Bilal RM, Batool F, Yameen K, Farag MR, Madkour M, Elnesr SS, El-Shall NA, Dhama K, Alagawany M (2023). Application of herbs and their derivatives in broiler chickens: a review. World Poult Sci J.

[bib1.bib53] Reda FM, Rudayni HA, Mahmoud HK, El-Shall NA, Alawam AS, Allam AA, Farag MR, Alagawany M, Khairunnesa M (2025). *Ziziphus spina-christi* (L.) leaf extract improves growth, feed utilization, digestive enzymes and blood metabolites in broiler chickens. Vet Med Sci.

[bib1.bib54] Reitman S, Frankel S (1957). A colorimetric method for the determination of serum glutamic oxalacetic and glutamic pyruvic transaminases. Am J Clin Pathol.

[bib1.bib55] Reyer H, Zentek J, Männer K, Youssef IMI, Aumiller T, Weghuber J, Wimmers K, Mueller AS (2017). Possible molecular mechanism by which an essential oil blend from star anise, rosemary, thyme, and oregano and saponins increase the performance and ileal protein digestibility of growing broilers. J Agric Food Chem.

[bib1.bib56] Rotruck JT, Pope AL, Ganther HE, Swanson AB, Hafeman DG, Hoeskstra W (1973). Biochemical role as a component of glutathione peroxidase. Science.

[bib1.bib57] Saadat SH, Mozhgan M, Omidali E, Heshmatollah K (2016). effects of thymol and carvacrol on productive performance, antioxidant enzyme activity and certain blood metabolites in heat stressed broilers. Ir J Appl Anim Sci.

[bib1.bib58] Saleh AA, Hafez A, Amber K (2023). Drug-independent control strategy of clostridial infection in broiler chickens using anti-toxin environmentally friendly multienzymes. Sci Rep.

[bib1.bib59] Sang-Oh P, Chae-Min R, Byung-Sung P, Jong H (2013). The meat quality and growth performance in broiler chickens fed diet with cinnamon powder. J Environ Biol.

[bib1.bib60] SPSS (2008). Statistical package for the social sciences, Release 16.

[bib1.bib61] Stanaćev V, Glamočić D, Milošević N, puvača N, Stanaćev V, Plavša N (2011). Effect of garlic (*Allium sativum* L.) in fattening chicks nutrition. Afr J Agri Res.

[bib1.bib62] Van Oosten MJ, Pepe O, De Pascale S, Silletti S, Maggio A (2017). The role of biostimulants and bioeffectors as alleviators of abiotic stress in crop plants. Chem Biol Technol Agric.

[bib1.bib63] Wagner DD, Furrow RD, Bradley BD (1983). Sub chronic toxicity of growth promoters in broilers chickens. Vet Pathol.

[bib1.bib64] Windisch W, Schedle K, Plitzner C, Kroismayr A (2008). Use of phytogenic products as feed additives for swine and poultry. J Anim Sci.

[bib1.bib65] Yagi K (1984). Assay for blood plasma or serum. Methods Enzymol.

[bib1.bib66] Yan L, An S, Lv Z, Wang Z, Wu Y, Zhu Y, Zhao M, Sun C, Lv M, Zhu Z, Gu Y (2019). Effects of phytonutrients on growth performance, antioxidative status, and energy utilization of broilers fed low energy diets. Anim Nutrit.

